# MicroRNA-132-3p suppresses type I IFN response through targeting IRF1 to facilitate H1N1 influenza A virus infection

**DOI:** 10.1042/BSR20192769

**Published:** 2019-12-10

**Authors:** Fangyi Zhang, Xuefeng Lin, Xiaodong Yang, Guangjian Lu, Qunmei Zhang, Chunxiao Zhang

**Affiliations:** 1 Clinical Lab, Yueqing Hospital Affiliated to Wenzhou Medical University, Yueqing 325600, Zhejiang, China; 2 Clinical Lab, The First Affiliated Hospital of Xinxiang Medical University, Weihui 453100, Henan, China; 3 Blood Transfusion Room, The First Affiliated Hospital of Xinxiang Medical University, Weihui 453100, Henan, China; 4 Respiratory Intensive Care Unit, The First Affiliated Hospital of Xinxiang Medical University, Weihui 453100, Henan, China

**Keywords:** H1N1 IAV, miR-132-3p, replication, IRF1, Type I IFN

## Abstract

Increasing evidence has indicated that microRNAs (miRNAs) have essential roles in innate immune responses to various viral infections; however, the role of miRNAs in H1N1 influenza A virus (IAV) infection is still unclear. The present study aimed to elucidate the role and mechanism of miRNAs in IAV replication *in vitro*. Using a microarray assay, we analyzed the expression profiles of miRNAs in peripheral blood from IAV patients. It was found that miR-132-3p was significantly up-regulated in peripheral blood samples from IAV patients. It was also observed that IAV infection up-regulated the expression of miR-132-3p in a dose- and time-dependent manner. Subsequently, we investigated miR-132-3p function and found that up-regulation of miR-132-3p promoted IAV replication, whereas knockdown of miR-132-3p repressed replication. Meanwhile, overexpression of miR-132-3p could inhibit IAV triggered INF-α and INF-β production and IFN-stimulated gene (ISG) expression, including myxovirus protein A (MxA), 2′,5′-oligoadenylate synthetases (OAS), and double-stranded RNA-dependent protein kinase (PKR), while inhibition of miR-132-3p enhanced IAV triggered these effects. Of note, interferon regulatory factor 1 (IRF1), a well-known regulator of the type I IFN response, was identified as a direct target of miR-132-3p during HIN1 IAV infection. Furthermore, knockdown of IRF1 by si-IRF1 reversed the promoting effects of miR-132-3p inhibition on type I IFN response. Taken together, up-regulation of miR-132-3p promotes IAV replication by suppressing type I IFN response through its target gene IRF1, suggesting that miR-132-3p could represent a novel potential therapeutic target of IAV treatment.

## Introduction

Influenza A virus (IAV) is a common human respiratory pathogen, which can cause both respiratory and constitutional effects [1]. The fatality rate caused by IAV gradually increased in most countries worldwide in recently years. During the virus infection, type I interferon (IFNα/β)-mediated immune response effectively prevents the replication of IAV [2]. However, the detailed mechanisms remain poorly characterized.

MicroRNAs (miRNAs) are a small conserved non-coding RNAs (∼21 nucleotides in length), which suppress gene expression through either inducing transcript degradation or inhibiting translation [3,4]. Previous studies have demonstrated the involvement of miRNAs in many viral infections, acting as regulators of anti-viral immune response [5–7]. For example, miR-373 was found to inhibit the replication of herpes simplex virus type 1 (HSV-1) through promotion of type I IFN response [8]. He et al. found that miR-182 inhibited human cytomegalovirus (HCMV) replication by targeting FOXO3 in neural cells [9]. In terms of IAV, several miRNAs have been described and controlled the IAV infection through several different mechanisms, such as miR-323, miR-491, and miR-654 [10]. However, the roles and mechanisms of miRNAs during AIV infection remain relatively unexplored.

In the present study, the miRNA expression profile was examined in peripheral blood of H1N1 IAV patients using microarray assay. Furthermore, the function and mechanisms of miR-132-3p in the immune response to AIV infection were investigated. Our findings provide a novel target for the treatment of IAV infection.

## Materials and methods

### Ethics statement and clinical specimens

Ten serum samples were obtained from patients with H1N1 influenza A virus at the Yueqing Hospital Affiliated to Wenzhou Medical University, Zhejiang, China between March and May 2018. Healthy controls were obtained randomly from individuals who had not been suffered from respiratory disease. Influenza patients recruited in the present study were confirmed as being either infected with H1N1 virus by conventional RT-qPCR with standard primers [11]. Peripheral blood samples were stored frozen at 20°C until analyzed. Participants’ information is summarized in Table 1 and no statistically significant difference was found between the influenza and control group for the age and gender distribution (*P* > 0.05). Three samples of each group were selected for the miRNA microarray analysis. The protocol was approved by Research Ethics Committee of Wenzhou Medical University. All test subjects provided written informed consent prior to participating in this study, which was approved by our university’s Institutional Review Board.

**Table 1:** Basic characteristics of healthy controls and H1N1 patients enrolled in the study

Sample characteristics	Healthy control	Patients
Number	13	13
Sex (male/female)	8/5	7/6
Age (mean ± SD)	27.34 ± 12.35	29.60 ± 11.31
Infectious diseases	None	H1N1 only
Onset clinical symptoms	None	Fever/cough

### Cell culture, virus and antibodies

A549 cells were purchased from American Type Culture Collection (ATCC, Manassas, VA, U.S.A.) and cultured in minimum Eagle’s medium (Gibco) supplemented with 10% fetal bovine serum (FBS, Hyclone) at 37°C in a humidified atmosphere of 5% CO_2_. Influenza virus A/Jingfang/01/1986(H1N1) strain was prepared in 11-day-old embryonated eggs. The prepared viruses were used to infect A549 cells. After incubation for 1 h, the cells were washed three times with PBS, and then infection medium was added to the cells. The infected cells were cultured at 37°C in 5% CO_2_. Mouse anti-M1 mAb and rabbit anti-NP polyclonal antibody were kindly provided by Wenjun Liu (Institute of Microbiology, Chinese Academy of Sciences). Anti-IRF1 was obtained from Abcam, Cambridge, MA, U.S.A.

### IAV infection

A549 cells (5 × 10^4^ cells/well) were seeded in six-well plates for 24 h. Prior to infection, PBS was used to wash the cells three times, and then virus diluted in serum-free MEM was added to each well. After incubation for 60 min, the cells were washed with PBS, and then fresh MEM media supplemented with 1 μg/ml of TPCK-trypsin (Sigma-Aldrich, St Louis, MO) was added and incubated at 37°C and 5% CO_2_.

### Plaque assay

The titers of IAV in culture media were measured using the plaque assays in A549 cells. Briefly, A549 cells (5 × 10^4^ cells/well) in six-well plates (Thermo Fisher Scientific, Australia) were infected with serial dilutions of H1N1 for 45 min at 37°C. Following this, 3 ml of overlay media containing 1% low-melting-point agarose (Sigma-Aldrich), 1 μg TPCK trypsin/ml and 100 U/ml Penicillin/Streptomycin (Life Technologies, Australia) were added to the wells. Plates were incubated at 37°C, 5% CO_2_ for 72 h and then were fixed with 4% paraformaldehyde for 30 min. The cells were stained with Crystal Violet (0.1% in 20% ethanol) to visualize. Then, the visible plaques were counted and virus titers were determined by counting the PFU (plaques) for each sample and expressed as PFU/ml.

### MicroRNA microarray analysis

RNA was extracted from peripheral blood samples from three influenza patients and three healthy controls using an miRNeasy Mini Kit (QIAGEN GmbH, Hilden, Germany) and then the RNA concentration were analyzed with an NanoDrop ND-2000 spectrophotometry (Thermo Fisher Scientific, Inc., Waltham, MA, U.S.A.). About 1 μg of total RNA was used as the input for the labeling reaction and hybridized using the miRCURY Hy3/Hy5 Power Labeling kit and miRCURY™ LNA array (v.16.0; Exiqon A/S, Copenhagen, Denmark) according to the manufacturer’s protocol. Data were analyzed using the ImaGene® 9 (miRCURY LNA™ microRNA Array Analysis Software, Exiqon). The raw intensity data were further analyzed using GeneSpring GX, version 7.3 (Agilent Technologies, Inc.).

### Quantitative reverse transcription (qRT)-PCR

Total RNA was isolated from peripheral blood samples or cells with TRIzol reagent (Invitrogen, Carlsbad, CA, United States). cDNA was synthesized using PrimeScript RT Master Mix (Takara, Tokyo, Japan), according to manufacturer’s instructions. Real-time PCR for miRNA and mRNA were performed using a standard protocol from the SYBR Green PCR kit (Toyobo, Osaka, Japan) on an ABI 7900HT Fast Real-Time PCR System (Life Technologies, U.S.A.). Relative quantification was determined by normalization to U6 or GAPDH. The primers for qRT-PCR analysis were as follows: miR-132-3p forward: 5′-GCGCGCGTAACAGTCTACAGC-3′; miR-132-3p reverse: 5′-GCGCGCGTAACAGTCTACAGC-3′; miR-126 forward: 5′-CCCAAGCTTAGTTATTGCTGCCCAGTTGC-3′. miR-126 reverse: 5′-GGACTAGTAAGGTAGGGAGGGGTGTTTCT-3′ miR-486 forward: 5′-GCCCTATTAACGCTGGCTTG-3′, miR-486 reverse, 5′-GTCAGATAGGGGCAGCGGTT-3′; miR-574 forward:5′-TCTGAGTGTGTGTGTGTG-3′, miR-574 reverse: 5′-GACTGTTCCTCTCTTCCTC-3′; miR-7 forward: 5′-CTAGCTAGCTAGAGCACCAATAGGGAAGGG-3′, miR-7 reverse 5′-GAAGATCTTCGAGTCTGCCGATGGGTGT-3′. U6 forward: 5′-TGCGGGTGCTCGCTTCGCAGC-3′; U6 reverse: 5′-CCAGTGCAGGGTCCGAGGT-3′; OAS forward: 5′-AGGTGGTAAAGGGTGGCT-3′, OAS reverse: 5′-TGCTTGACTAGGCGGATG-3′ MxA forward: 5′-GGGAAGGTGAAGGTCGGAGT-3′, MxA reverse: 5′-TTGAGGTCAATGAAGGGGTCA-3′; PKR forward: 5′-AGAGTAACCGTTGGTGACATAACCT-3′, PKR reverse: 5′-GCAGCCTCTGCAGCTCTATGTT-3′; GAPDH forward: 5′-AGGTCGGTGTGAACGGATTTG-3′, GAPDH reverse: 5′-TGTAGACCATGTAGTTGAGGTCA-3′. Relative quantities were calculated by the 2^−ΔΔ^Ct method.

### Transfection

The miR-132-3p mimics, mimics negative control (mimics NC), miR-132-3p inhibitor and inhibitor NC were bought from GenePharm (Shanghai, China). Non-specific siRNA (si-NC) and si-IRF1 were purchased from Invitrogen. Transfections of the miRNAs or si-IRF1 were performed by using Lipofectamine 2000 (Invitrogen, Thermo Fisher Scientific, Inc.) according to the manufacturer’s instructions. After 24 h the transfection, the cells were infected with H1N1 virus at MOI = 1.

### ELISA

Cell culture supernatants were collected 12 h after H1N1 virus infection. Secreted IFN-α and IFN-β levels in the cell supernatants were determined with human Interferon-α ELISA kit (Dakewe, Shenzhen, China) and human IFN-β ELISA kit (PBL interferon source, U.S.A.).

### Immunofluorescence

After 24 h the transfection, the cells were infected with virus for another 12 h, and then fixed in absolute ethyl alcohol for 15 min at room temperature, washed twice with PBS. Fixed cells were stained with primary antibody (anti-M1, 1:200 dilutions) for 1 h at room temperature. After incubation with secondary antibody conjugated with FITC (1:100, Sigma-Aldrich, St Louis, MO) for 2 h in the dark, fluorescence images were collected and analyzed using an inverted fluorescence microscope.

### Dual luciferase activity assay

WT or mutant of 3′UTR sequences of IRF1 were amplified and cloned into the pGL3 vector (Promega, Madison, WI, U.S.A.). A549 cells were seeded in 96-well plates and co-transfected with these vectors and miR-132-3p mimics, mimics NC, miR-132-3p inhibitor and inhibitor NC. At 24 h post-transfection, the luciferase activity was determined by Dual Luciferase Reporter Assay System (Promega).

### Western blot

The total protein from the cells was isolated using a radioimmunoprecipitation assay buffer (Beyotime Biotechnology, Shanghai, China) with a protease inhibitor cocktail (Pierce Protein Biology, Rockford, IL, U.S.A.). The protein concentration was determined using a BCA protein assay kit (Pierce, Rockford, IL, U.S.A.). A sample of 20 μg proteins was separated on SDS-PAGE gel and then transferred onto polyvinylidene difluoride membranes (GE Healthcare, Freiburg, DE). The membranes were blocked with 5% nonfat milk and incubated overnight at 4°C with primary antibody against IRF1 (Cat. no #8478, Cell Signaling Technology, Danvers, MA, 1:2000 dilution), Mouse anti-M1 mAb and rabbit anti-NP polyclonal antibody (1:2000 dilution) and β-actin (Cat. no #3700, Cell Signaling Technology, Danvers, MA, 1:2000 dilution) were probed with proteins on the membrane at 4°C overnight. After incubating with secondary antibodies (Cat. #14709; Cell Signaling Technology, Danvers, MA, 1:10,000 dilution), bands were detected by enhanced chemiluminescence (ECL) kit (GE Healthcare, Freiburg, DE), and the bands intensity was analyzed by Image J software (Rawak Software, Inc. Munich, Germany).

### Statistical analysis

All of the analyses were performed using the SPSS program (version 18.0; SPSS, Chicago, IL, U.S.A.). Differences between two groups were analyzed using Student’s *t*-test and for multiple groups one way ANOVA followed by Tukey’s post hoc test were used. Numerical data are presented as the mean ± SD; *P* < 0.05 was defined as significant, and *P* < 0.01 was defined as very significant.

## Results

### miR-132-3p was up-regulated by H1N1 IAV infection

**Figure 1:**
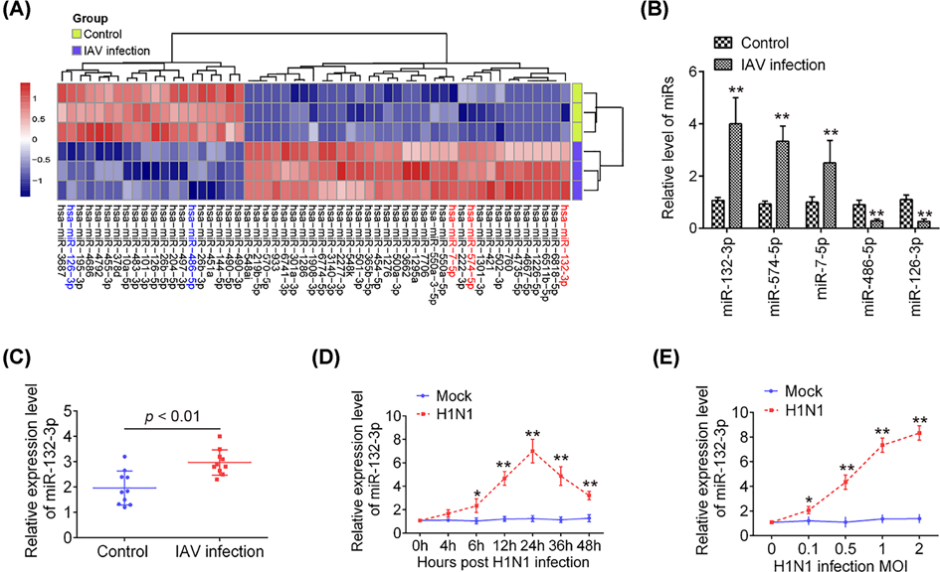
miR-132-3p was up-regulated during IAV infection (**A**) Heatmap of normalized expression levels of miRNAs in peripheral blood samples from IAV patients and healthy controls (*n* = 3). Blue indicates low expression levels; red indicates high expression levels. (**B**) Peripheral blood samples from patients with IAV and healthy persons were collected and miR-132-3p, miR-126, miR-486, miR-574 and miR-7 expression levels were detected by qRT-PCR analysis (*n* = 3); *P*  <  0.01 versus Control group. (**C**) miR-132-3p expression levels were detected by qRT-PCR in peripheral blood samples from patients with IAV and healthy persons analysis (*n* = 10). (**D** and **E**) A549 cells were infected with IAV either at indicated time at a MOI of 1 (D) or at indicated MOIs for 24 h (E), and then the cells were harvested for further qRT-PCR analysis of miR-132-3p expression. Data are presented as means of three independent experiments ± SD; *P  <  0.05, **P  <  0.01 versus Mock group.

Previous studies have reported the involvement of microRNAs in antiviral responses of host cells to many viruses [12–14]. Peripheral blood samples were collected from controls and patients with IAV infection for miRNA microarray and qRT-PCR studies. As shown in Table 1, no statistically significant difference was found between the influenza and control group for the age and gender distribution. Microarray analysis was used to determine miRNA expression levels in peripheral blood samples from H1N1 IAV infected patients and healthy controls. Compared with the control group, a total of 35 miRNAs were up-regulated and 20 miRNAs were down-regulated in patients infected with H1N1 IAV (Figure 1A). For miR-126, miR-132-3p and miR-486 have been reported to be up-regulated, miR-7 and miR-574 were down-regulated in IAV infection progression [15–18]. We verified the expressional patterns of the five microRNAs by qPCR analysis indicating the reliability of our microarray. MiR-132-3p was the mostly up-regulated miRNA in patients infected with H1N1 IAV and selected for further analysis (Figure 1B). It has previously been shown that miR-132-3p is highly expressed following infection with herpes simplex virus-1 (HSV-1), and human cytomegalovirus (HCMV), and that miR-132 regulates innate antiviral immunity by inhibiting expression of the p300 transcriptional co-activator [19]. A recent study has demonstrated that miR-132 was also highly up-regulated in response to infection with HIV-1 and enhanced HIV-1 replication [20]. It was also found that miR-132-3p was up-regulated after infection with IAV in human respiratory cells [18]. However, the roles of miR-132-3p in H1N1 IAV infection remain unknown. To validate the expression of miR-132-3p, we further measured the expression of miR-132-3p in ten peripheral blood samples from H1N1 IAV infected patients by qRT-PCR. As shown in Figure 1C, miR-132-3p was significantly up-regulated in patients infected with H1N1 IAV compared with the control group. Furthermore, we detected the expression levels of miR-132-3p in A549 cells infected with H1N1 IAV. miR-132-3p expression was dramatically increased upon IAV infection and the up-regulation of miR-132-3p levels showed a dose-dependent manner (Figure 1D). Next, we measured miR-132-3p levels at different time points of IAV infection. The up-regulation of miR-132-3p levels upon IAV infection also showed a time-dependent manner (Figure 1E). Collectively, our data suggest miR-132-3p may play a part in IAV infection.

### miR-132-3p regulated IAV replication

**Figure 2:**
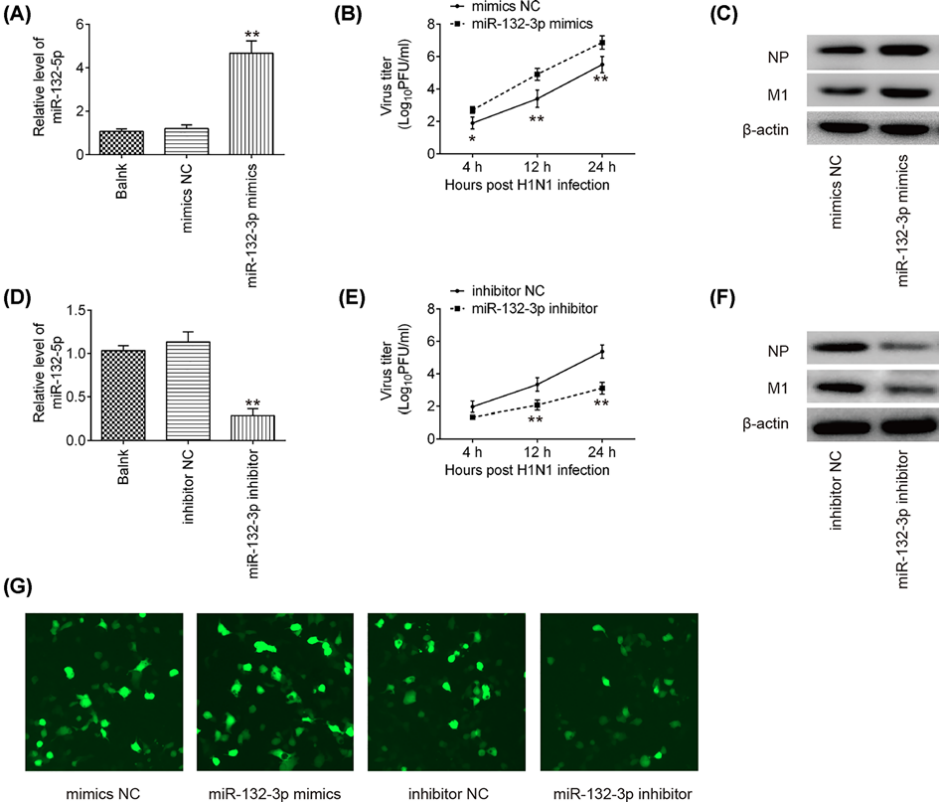
miR-132-3p promoted IAV replication A549 cells were transfected with miR-132-3p inhibitor, inhibitor NC, miR-132-3p mimics and mimics NC. Twenty-four hours post-transfection, cells were infected with IAV at MOI = 1. (**A** and **D**) The transfection efficiency of miR-132-3p mimics and inhibitor were determined by qRT-PCR. Data rare presented as means of three independent experiments ± SD. **P < 0.01 versus mimics NC group or inhibitor NC group. (**B** and** E**) The viral titers in the cell cultures were determined by plaque assay using six-well plates. Data are presented as means of three independent experiments ± SD; *P < 0.05, **P < 0.01 versus mimics NC or inhibitor NC group. (**C** and** F**) Levels of M1 and NP protein expression were determined by Western blot assay. (**G**) Levels of M1 protein expression were detected by immunofluorescence.

To investigate whether miR-132-3p affects IAV replication, miR-132-3p mimics or miR-132-3p inhibitor were transfected into A549 cells, followed by IAV infection. The expression levels of miR-132-3p were notably increased or decreased after miR-132-3p mimics or miR-132-3p inhibitor transfection (Figure 2A,D). Subsequently, plaque assay and Western Blot assays were performed to examine their effects on IAV replication. As shown in Figure 2B,E, overexpression of miR-132-3p significantly resulted in significant increases in viral titers of IAV compared with that in mimics NC–transfected A549 cells, while knockdown of miR-132-3p inhibited viral titers of IAV compared with that inhibitor NC-transfected A549 cells. Nucleoprotein (NP) and matrix protein (M1) are the most abundant protein in the IAV viral particle [21]. Several lines of evidence have shown that certain miRNAs could affect viral replication through regulation of these IAV viral proteins [22,23]. Therefore, we determined whether the expressions of viral protein M1 and NP were affected by miR-132-3p. As shown in Figure 2C,F, the expressions of viral protein M1 and NP were obviously increased when miR-132-3p was overexpressed, whereas they were substantially decreased after miR-132-3p was knocked down in IAV infected A549 cells. Consistently, the expression of M1 was found to be increased or decreased in miR-132-3p mimics or miR-132-3p inhibitor transfected A549 cells, as examined by immunofluorescence (Figure 2G). These data indicate that miR-132-3p up-regulation can promote IAV replication, whereas miR-146a down-regulation inhibited IAV replication.

### miR-132-3p negatively regulates IAV-triggered type I IFN production in A549 cells

**Figure 3:**
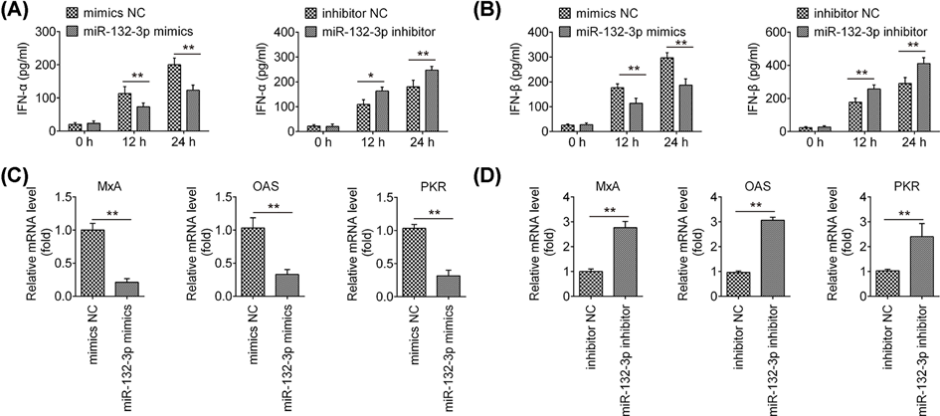
miR-132-3p regulates IAV-triggered type I IFN production in A549 cells A549 cells were transfected with miR-132-3p inhibitor, inhibitor NC, miR-132-3p mimics and mimics NC. Twenty-four hours post-transfection, cells were infected with IAV at MOI = 1. (**A** and **B**) Cell and supernatant were harvested at 0, 12 and 24 h post-infection, and then ELISA assay were performed to measure IFN-α and IFN-β expression. (**C** and **D**) qRT-PCR assay were performed to measure ISGs expression (MxA, OAS and PKR). Data are presented as means of three independent experiments ± SD; *P < 0.05, **P < 0.01 versus mimics NC or inhibitor NC group.

During the IAV infection, innate antiviral mechanisms dominated by type I interferon are potentially the most important pathways of the host defense against IAV replication [24]. We further explore the effect of miR-132-3p on the regulation of IAV-triggered immune response. Our results showed that overexpression of miR-132-3p reduced the expressions of IFN-α and IFN-β, while inhibition of miR-132-3p enhanced the expressions of IFN-α and IFN-β in A549 cells in response to IAV infection (Figure 3A,B). It was also observed that overexpression of miR-132-3p inhibited the expressions of conventional interferon stimulated genes (ISGs), including MxA, OAS and PKR, whereas miR-132-3p inhibition significantly promoted the expressions of these ISGs. These data suggest that miR-132-3p negatively regulates IAV-triggered type I IFN response in A549 cells.

### IRF1 was a direct target of miR-132-3p

**Figure 4:**
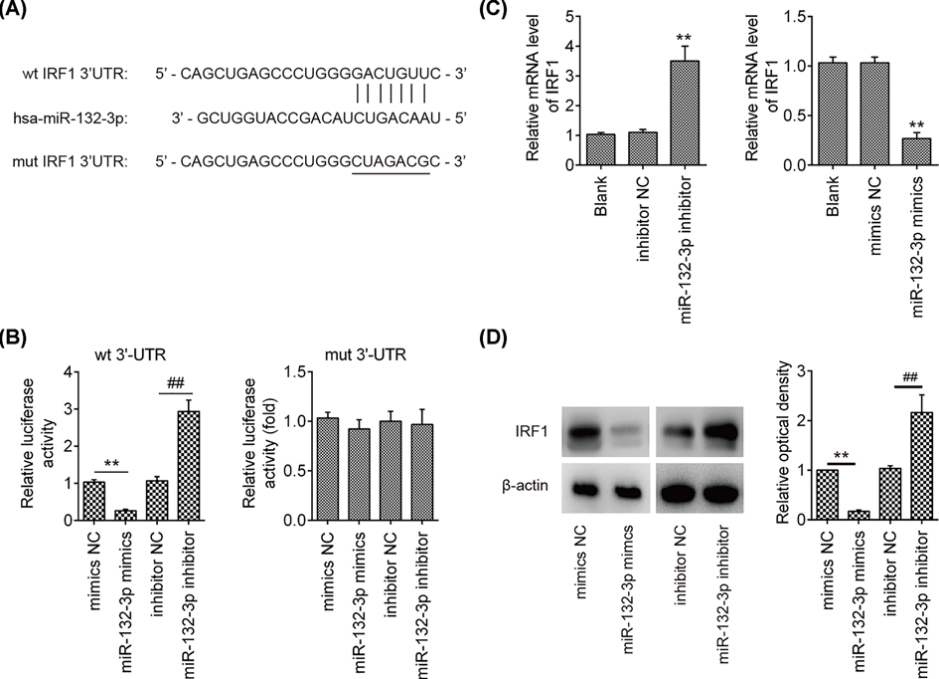
IRF1 is a direct target of miR-132-3p (**A**) Putative binding site of miR-132-3p and IRF1 with mut and wt 3′UTRs. (**B**) Luciferase assay of A549 cells co-transfected with firefly luciferase constructs containing the IRF1 wild-type or mutated 3′UTRs and miR-132-3p mimics, mimics NC, miR-132-3p inhibitor or inhibitor NC, as indicated (*n* = 3). Data are presented as means of three independent experiments ± SD; **P < 0.01 versus mimics NC or inhibitor NC. (**C** and **D**) The expression of IRF1 mRNA and protein after transfection with miR-132-3p mimic or miR-132-3p inhibitor were measured by qRT-PCR and Western Blot. Data rare presented as means of three independent experiments ± SD; **P < 0.01 versus mimics NC, ^##^*P* < 0.01 versus inhibitor NC group.

To explore the molecular mechanism by which miR-132-3p modulates type I IFNs and viral replication, two publicly available databases, miRanda and Targetscan, were used to predicate the potential targets of the miR-132-3p that could regulate type I IFNs and viral replication. Bioinformatics analyses showed that interferon regulatory factor 1 (IRF1) was considered as one of the candidates after analysis. It has previously been reported that IRF1 can bind to the promoter of IFN-β and activate the expression of IFN-β [25]. As shown in Figure 4A, the complementary sequence of miR-132-3p was found in the 3′UTR of IRF1 mRNA. To validate whether IRF1 directly target IRF1, a luciferase reporter assay was performed. The results showed that miR-132-3p mimics significantly repressed luciferase activity in A549 cells, while miR-132-3p inhibitor significantly enhanced luciferase activities of the IRF1 3′UTR segment compared with that in the NC group; however, these effects were abrogated in those of the construct containing a mutant binding site (IRF1 3′UTR-mut) (Figure 4B). qRT-PCR and Western Blot assays showed that the mRNA and protein expression of IRF1 was significantly suppressed by miR-132-3p mimics, while markedly promoted by miR-132-3p inhibitor compared with that in NC group (Figure 4C,D). These findings indicated that IRF1 might be a functional target of miR-132-3p.

### Knockdown of IRF1 reversed the inhibitory effects of miR-132-3p inhibition on IAV replication and type I IFN production

**Figure 5:**
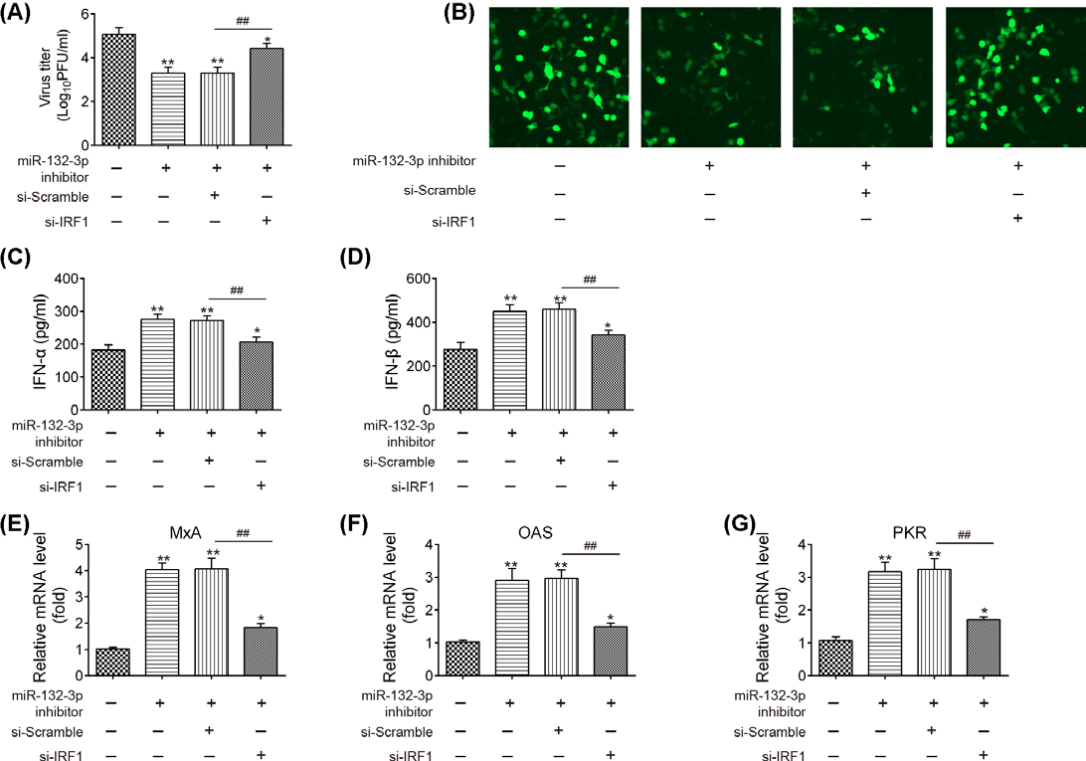
MiR-132-3p promotes IAV replication by targeting IRF1 A549 cells were transfected with miR-132-3p inhibitor, si-IRF1, or both. Twenty-four hours after transfection, cells were infected with IAV at MOI = 1. (**A**) The viral titers in the cell cultures were determined by plaque assay using six-well plates. (**B**) Levels of M1 protein expression were detected by immunofluorescence. (**C** and **D**) ELISA assay were performed to measure IFN-α and IFNβ expression. (**E**–**G**) qRT-PCR assay were performed to measure ISGs expression (MxA, OAS and PKR). Data rare presented as means of three independent experiments ± SD. *P < 0.05, **P < 0.01 versus control group, ^##^*P* < 0.01 versus miR-132-3p inhibitor + si-Scramble group.

To further investigate whether miR-132-3p inhibition suppressed the IAV replication by inducing IRF1, si-IRF1 and miR-132-3p inhibitor were co-transfected into A549 cell 24 h prior to IAV infection, and incubated for 12 h. It was observed that the virus titers and the expression of M1 protein were significantly decreased in the A549 cells following miR-132-3p inhibitor, compared with that in the IAV infection group, whereas these inhibitory effects of miR-132-3p inhibitor were attenuated by the knockdown of IRF1 (Figure 5A,B). We also assessed the effects of IRF1 inhibition on the expressions of IFN-α and IFN-β, as well as the expression of ISGs in miR-132-3p inhibitor transfected A549 cells. The results showed that IRF1 knockdown partially reversed the inhibitory effects of miR-132-3p inhibitor on the expressions of IFN-α, IFN-β, MxA, OAS and PKR, suggesting that miR-132-3p inhibition suppressed IAV replication by promoting the expression of IRF1 (Figure 5C–G).

## Discussion

In the present study, miR-132-3p was found to be significantly up-regulated during IAV infection. Moreover, we further demonstrated that miR-132-3p alleviated the type I IFN-mediated antiviral defense by targeting IRF1, and thereby promoting IAV replication. Our findings suggest that miR-132-3p may be a potential therapeutic target in IAV infection.

Increasing evidences have shown the involvement of several miRNAs in host defense against viral infections including IAV [26,27]. For example, Hu et al. showed that miR-33a up-regulation suppressed H1N1 virus replication by directly binding to the 3′UTR of Archain 1 (ARCN1) RNA [28]. miR-194 was found to suppress the expression of FGF2 that is a novel antiviral regulator, thus inhibiting IAV replication [29]. Ingle H et al. found that miR-485 was produced in response to viral infection and inhibition of miR-485 markedly reduced the replication of IAV in mammalian cells [30]. This information suggests the important roles of miRNAs in host defense against IAV infections. In the present study, using a microarray analysis, we observed that large numbers of miRNAs were significantly deregulated in the peripheral blood from IAV patients; in particular, miR-132-3p attracted our attention as its expression was one of the most being up-regulated miRNAs during IAV infection. Previous studies have reported that miR-26a acted as a regulator of the human immunodeficiency virus (HIV) and HCMV infection [19,20]; however, whether miR-132-3p affects IAV infection is still unclear. In our study, we found that overexpression of miR-132-3p greatly suppressed IAV replication, while inhibition of miR-132-3p promoted IAV replication, as evidence by titers of virus. It was also found that overexpression of miR-132-3p inhibited Type I IFN production, INF-α and INF-β, as well as the expressions of ISGs including MxA, OAS and PKR in IAV, whereas inhibition of miR-132-3p promoted the expression of INF-α and INF-β and these ISGs. These data indicated that miR-132-3p contribute to through negatively modulation of Type I IFN response.

IRF1 is known as a key transcription activator of the Type I IFN response [31]. Inhibition of IRF1 could dramatically increase the cellular susceptibility to kinds of viruses, such as hepatitis C virus [32] and HSV-1 [8]. Kuriakose et al. demonstrated the role of IRF1 in promoting NLRP3 inflammasome activation and ZBP1-induced cell death during IAV infection [33]. In addition, it has previously reported that Type I IFNs were suppressed by miRNAs through targeting IRF1 during HSV-1 [8] and porcine reproductive and respiratory syndrome virus (PRRSV) infection [34]. However, whether IRF1 is a functional target of miR-132-3p during IAV infection remained to be elucidated. In our study, we identified IRF1 was a direct target of miR-132-3p during H1N1 IAV infection. Moreover, the inhibitory effect of miR-132-3p knockdown on IAV replication was abrogated by IRF1 inhibition, suggesting that miR-132-3p inhibitor suppressed IAV replication via directly targeting IRF1, thus enhancing the antiviral response of host.

There are several limitations in our study. All these results obtained from *in vitro*, thus, we will performed influenza virus challenge experiments *in vivo* to test whether inhibition of miR-132-3p by antagomir-132-3p injection has a protective role during IAV infection in mice. In another aspect, we will confirm the role of miR-132-3p in the airway epithelial cells, such as primary murine tracheal epithelial cells and mouse alveolar macrophages (RAW264.7) that serve as the first and overwhelmingly primary target for virus infection and growth [35,36]. In this connection, more basic researches will provide us a better understanding in this regard.

In conclusion, we demonstrated that miR-132-3p facilitates IAV replication in A549 cells by targeting IRF1 to suppress Type I IFN response. These findings provide new insight on the role of host miRNAs in AIV infection and suggest that miR-132-3p may be an important therapy target for the prevention and control of IAV.

## Abbreviations

ARCN1: Archain 1
HCMV: human cytomegalovirus
HIV: human immunodeficiency virus
HSV-1: herpes simplex virus-1
IAV: influenza A virus
IRF1: interferon regulatory factor 1
ISG: interferon stimulated gene
M1: matrix protein
MxA: myxovirus protein A
NP: nucleoprotein
OAS: 2′,5′-oligoadenylate synthetases
